# Associative nitrogen fixation in nodules of the conifer *Lepidothamnus fonkii (Podocarpaceae)* inhabiting ombrotrophic bogs in southern Patagonia

**DOI:** 10.1038/srep39072

**Published:** 2016-12-15

**Authors:** Werner Borken, Marcus A. Horn, Stefan Geimer, Nelson A. Bahamonde Aguilar, Klaus-Holger Knorr

**Affiliations:** 1 University of Bayreuth, Department of Soil Ecology, Dr.-Hans-Frisch-Str. 1-3, 95448 Bayreuth, Germany; 2 University of Bayreuth, Ecological Microbiology, Dr.-Hans-Frisch-Str. 1-3, 95448 Bayreuth, Germany; 3 University of Hannover, Institute of Microbiology, Herrenhäuserstr. 2, 30140 Hannover, Germany; 4 University of Bayreuth, Cell Biology and Electron Microscopy, Universitätsstr, 30, 95440 Bayreuth, Germany; 5 Universidad de Magellanes, Instituto de la Patagonia, Laboratorio de Botanica, Av. Manuel Bulnes 01890, Punta Arenas, Chile; 6 University of Münster, ILÖK, Hydrology group, Heisenbergstr, 2, 48149, Münster, Germany; 7 ONG AMA Torres del Paine, Estancia Cerro Paine S/N, Torres del Payne, Chile

## Abstract

Biological N_2_ fixation (BNF) in the rhizosphere of *Podocarpaceae* is currently attributed to unspecific diazotrophs with negligible impact on N acquisition. Here, we report specific and high associative BNF in dead cells of root nodules of *Lepidothamnus fonkii* distributed in ombrotrophic peatlands of Patagonia. BNF of nodulated roots, intact plants of *L. fonkii* and rhizospheric peat was assessed by ^15^N_2_ and acetylene reduction. Diazotrophs were identified by electron microscopy, analysis of nitrogenase encoding genes (*nifH*) and transcripts, and 16S rRNA. Nitrogenase encoding nifH transcripts from root nodules point to *Beijerinckiaceae* (*Rhizobiales*), known as free-living diazotrophs. Electron microscopy and 16S rRNA analysis likewise identified active *Beijerinckiaceae* in outer dead cells of root nodules. *NifH* transcripts from the rhizopshere peat revealed diverse active diazotrophs including *Beijerinckiaceae*. Both methods revealed high activity of nitrogenase rates in cut roots of *L. fonkii* (2.5 μmol N g^−1^ d.w. d^−1^ based on ^15^N_2_ assay; 2.4 μmol C_2_H_4_ g^−1^ d.w. d^−1^ based on acetylene reduction assay). The data suggest that (i) nodules recruit diazotrophic *Beijerinckiaceae* from peat, (ii) dead nodule cells provide an exclusive habitat for *Beijerinckiaceae*, and (iii) BNF in *L. fonkii* is one potent pathway to overcome N deficiency in ombrotrophic peatlands of Patagonia.

Biological dinitrogen (N_2_) fixation (BNF) by plant-associated prokaryotes is a widespread and effective process of N acquisition^1^. However, the capability of plants to host N_2_ fixing endosymbiotic prokaryotes is restricted to few plant species and bacteria^2^. Especially in peatlands, the existence of such mutualistic associations is restricted to very few plant species. One example is the shrub *Myrica gale* (L.) that grows in some peatlands of the Northern hemisphere and fixes considerably amounts of atmospheric N_2_ in root nodules^3^. Mutualistic BNF in peatlands of the southern hemisphere is not known, but other strategies of BNF can also occur in peatlands.

Conifers of the family *Podocarpaceae* form nodules and host arbuscular mycorrhizal fungi therein as shown for few species^4^,^5^. BNF in root nodules of *Podocarpaceae* has been postulated in several studies for more than a century^6^,^7^,^8^,^9^,^10^,^11^. Low N_2_ fixation activities in root nodules were confirmed for *Podocarpus rospigliosii*^6^ and *P. macrophyllus*^10^ (Table 1). In contrast, an absence of any N_2_ fixation was reported for *P. totara*^12^. The current view is that the observed N_2_ fixation activities arise from methodological artifacts and free-living diazotrophs in the rhizosphere, possibly due to incomplete removal of rhizosphere soil^11^,^13^.

*Podocarpaceae* are restricted to nutrient poor environments of the Southern Hemisphere^14^. Many nutrient poor *Sphagnum* bogs of Patagonia host the podocarp *Lepidothamnus fonkii* (Phil), a small coniferous shrub of up to 30–60 cm height with hitherto unknown associated N_2_ fixation (Fig. 1A). Pristine ombrotrophic bogs receive inorganic N from two sources: (i) atmospheric deposition and (ii) N_2_ fixation by non-symbiotic diazotrophic microorganisms^15^,^16^,^17^,^18^,^19^. Atmospheric N deposition is secondary relative to BNF as indicated by exceptionally low atmospheric deposition rates of less than 0.1 g N m^−2^ yr^−1 ^20 and BNF associated with *Sphagnum* or other mosses ranging from 0.5–6.4 g N m^−2^ yr^−1 ^21.

Net N retention rates and thus N storage in the peat of 2.0 g m^−2^ yr^−1^ reported for northern Alberta bogs were mainly attributed to BNF^17^. Patagonian bogs could have net N retention of 0.4–1.0 g N m^−2^ yr^−1^ based on published carbon (C) storage rates of 15–40 g C m^−2^ yr^−1^ in Yu *et al*.^22^ and average C/N ratios of about 40 in the top 1 m^19^,^23^. BNF thus likely represents the predominant N source (0.4–1.2 g N m^−2^ yr^−1^) in Patagonian bogs given the low N deposition. The widespread occurrence of *L. fonkii* in such habitats and the limited knowledge on BNF in *Podocarpaceae* nodules necessitates studies on the potential of *L. fonkii* for BNF. Thus, our objectives were to (i) assess associative N_2_ fixation in root nodules of *L. fonkii*, (ii) to identify the genetic potential for diazotrophy in root nodules as well as active diazotrophs, and to (iii) estimate the importance of *L. fonkii* for N acquisition in two pristine Patagonian bogs.

## Materials and Methods

**Figure 1:**
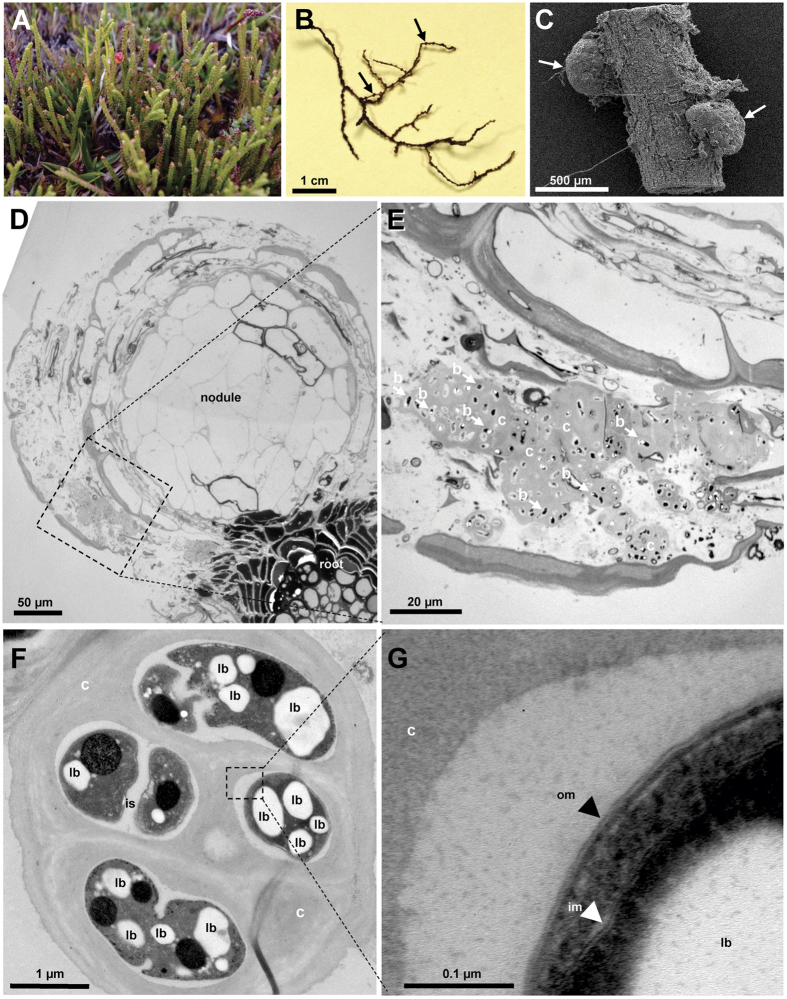
Photograph (**A**), stereo microscope image (**B**), scanning electron (SEM; **C**) and transmission electron microscope (TEM; **D**–**G**) images of *Lepidothamnus fonkii*. Photograph of *L. fonkii* at the SKY field site at Seno Skyring (Southern Patagonia, Chile; **A**) and roots densely covered by nodules (**B**). Root nodules (arrows) were smaller than 500 μm in diameter (SEM; **C**). Ultrastructure of root with nodule (TEM; **D**) revealed capsules with multiple bacteria located primarily at the vicinity of the nodules (arrows indicate some of the bacterial cells; **E**) Enlarged capsules indicate ultrastructure of bacteria containing lipoid bodies (**F**). Intact outer and inner membranes (black and white arrowheads, respectively; **G**) of bacteria were indicative of living gram negatives, which is in agreement with active diazotrophic *Beijerinckiaceae*-related bacteria detected at the roots. Bar represents a scale bar (**B**–**G**). Squares and dashed lines indicate areas that were enlarged in the following panel. Abbreviations: b, bacteria; c, capsule; im, inner membrane; is, intercellular space; lb, lipoid bodies; om, outer membrane.

Intact plants of *L. fonkii* (Fig. 1A) and peat cores of 100 cm^2^, 20 cm depth, with *L. fonkii* were sampled at two pristine bogs in Southern Patagonia, Chile, in March 2014: a 2–4 m deep peat deposit at the Seno Skyring (site ‘SKY’, −52.508667°S, 72.127278°W)^23^ and a 2 m deep peat deposit at the Seno Obstruccion (site ‘OBS’, 52.135907°S, 72.446037°W). Additional peat cores were obtained at each site using a Russian type peat corer (5 cm diameter), in which we determined peat C and N concentrations, C/N ratios, and δ^15^N signatures of the upper 80 cm (provided in the supporting information; Supplementary Table S1). *L. fonkii* grew in communities with *Astelia pumila (Asteliaceae), Donatia fascicularis (Stylidiaceae), Sphagnum magellanicum (Sphagnaceae), Empetrum rubrum (Ericaceae), Gaultheria pumila (Ericaceae), Drosera uniflora (Droseraceae), Marsippospermum grandiflorum (Juncaceae)*, and *Caltha appendiculata (Ranunculaceae)*. For estimates of plant biomass (n = 6) additional intact peat blocks were extruded (March 2013 and 2014). All samples were packed in plastic bags and transported to the lab. Leaf biomass, stems, and roots were manually picked, washed, dried, and weighed.

BNF was determined using both the ^15^N_2_ (98 atom%, Sigma Aldrich, batch No. MBBB0968V, St. Louis, USA) assay and the acetylene (Riessner-Gase, Lichtenfels, Germany) reduction assay (ARA)^24^. According to Dabundo *et al*.^25^ we cannot exclude that ^15^N_2_ was contaminated with reactive N gas compounds such as ^15^NH_3_ and ^15^NO_x_. A mean contamination as reported in Dabundo *et al*.^25^ (e.g. 1 mmol ^15^N mol^−1 15^N_2_) would overestimate the N_2_ fixation rates by about 13%, assuming an uptake of 50% of the total contamination. We assume that moist paper tissues in the jars (see below) trapped a large fraction of potential impurities during the incubation.

After washing and removal of peat particles, intact, non-sterilized plants were incubated in 325 ml glass jars. Roots were wrapped in moist paper tissue to prevent desiccation. For ^15^N_2_ assays, the jars (800 ml) were closed, evacuated to ~350 mbar and refilled with ^15^N_2_ to achieve about 70 atom% ^15^N_2_ in the head space (verified by mass spectrometry). In addition, freshly cut roots (0.1–0.2 g d.w.) and peat without live roots (about 0.1 g d.w.) were separately incubated in 22 ml vials with similar ^15^N_2_ enrichment in the headspace as for intact plant samples. In parallel batches all samples were incubated with 10 vol. % of acetylene in the headspace for the ARA. To mimic oxygen concentrations in the rhizosphere, we adjusted O_2_ concentrations to about 2%. Three replicates per site, each with 2–3 intact plants per jar were incubated for 65 hours at 15 °C in a climate chamber with 12 hours light per day (about 500 μmol s^−1^ photon flux). In the ARA, ethylene (C_2_H_4_) concentrations were analyzed after 0, 6, 12 and 24 hours (*L. fonkii* samples) or after 0, 1.5 and 5 h (*T. repens* controls), C_2_H_4_ production rates were obtained from linear increase of concentration over time and expressed in μmol C_2_H_4_ g^−1^ d.w. d^−1^. All ethylene time series were highly linear, with an r^2^ > 0.95. From ^15^N_2_ incubations, jars were opened and the plants were separated into roots, stems, and leaf biomass. After oven drying at 40 °C, the dry weight was determined and the material was milled for subsequent isotope analysis. In addition to intact plant incubations, three replicates per site of cut roots of *L. fonkii* were incubated separately in 20 ml flasks at 15 °C in the dark for 72 hours. As a control, parallel incubations of fresh, nodulated roots of *Trifolium repens* were carried out in triplicate. N_2_ fixation in the incubation was expressed in μmol N per gram dry biomass and day (μmol N g^−1^ d.w. d^−1^) and was calculated based on ^15^N natural abundance of control plants (Table S2) and ^15^N enrichment in atom % after incubation for each respective plant component. We are aware that some studies suggested to include only data from short term incubations for ARA and ^15^N_2_ techniques^26^,^27^, but recent studies in wetlands have confirmed linearity also over long incubation time (>48 h)^16^,^17^,^28^. Moreover, longer incubation times would also overcome at least in part methodological issues of an underestimation of rates due to incomplete equilibration of the added gas (acetylene or ^15^N_2_) and the water phase^29^ surrounding the samples of wetland plants.

Concentrations of ^15^N_2_ in the headspace were analyzed using a Delta Plus XL isotope ratio mass spectrometer (Thermo Finnigan, Bremen, Germany), after equilibration of the masses 28, 29, and 30 in a microwave (GMW 24–201, AHF Analysentechnik, Tübingen, Germany)^30^. Ethylene concentration was analyzed by gas chromatography (Model 8610C, SRI Instruments Inc., Las Vegas, NV, USA). Peat and plant analysis for ^15^N was done using a Eurovector/HEKAtech Elemental CNS analyzer (HEKAtech, Wegberg, Germany) coupled to a Nu Horizon isotope ratio mass spectrometer (Nu Instruments, Wrexham, UK).

Sections of *L. fonkii* roots were fixed in glutaraldehyde (2%) and OsO_4_ (2%) prior to positive staining with uranylaceate (2%) for ultrastructural analyses. For taking images with scanning electron microscopy (SEM, Philips ESEM XL 30), fixed sections were dehydrated in acetone, followed by critical point drying and sputtering with a gold layer. For transmission electron microscopy (TEM) (Zeiss CEM 902 or a JEOL JEM-2110) sections were dehydrated in ethanol/propylenoxid, embedded in epon, and thin sections (50–70 μM thickness) were produced in a Leica Ultracut UCT microtome.

Nitrogenase encoding genes (*nifH*) and transcripts, as well as 16S rRNA were analyzed from *L. fonkii* roots and peat to identify microbes driving N_2_ fixation and those colonizing roots. One mixed, representative sample of young densely nodulated roots from multiple individuals (600 mg) of *L. fonkii* were washed 2x with 70% ethanol and 3x with sterile phosphate buffered saline to remove root surface attached microbes prior to pestling and DNA/RNA extraction with the RNA PowerSoil and DNA Elution Accessory Kit (MoBio, Carlsbad, CA, USA). A representative, mixed sample of peat was extracted in a similar way. Reverse transcription was done with random hexamer priming and SuperScriptIII reverse transcriptase (Invitrogen, Karlsruhe, Germany) according to the manufacturer’s protocol^31^. *nifH* was PCR amplified from DNA and cDNA of roots and peat with primers IGK3 (GCI WTH TAY GGI AAR GGI GGI ATH GGI AA) and DVV (ATI GCR AAI CCI CCR CAI ACI ACR TC) currently covering the broadest range of *nifH* diversity (including *nifH* of Cyanobacteria) utilizing the Kappa2G Robust HotStart PCR Kit (Peqlab, Nuremberg, Germany) as described^32^. In brief, reaction mixtures contained 1x buffer A, 0.2 mM of each dNTP, 1.0 μM of each primer, 1x KAPPA Enhancer 1, 2.5 mM MgCl_2_, and 1 unit of KAPPA2G Robust HotStart DNA polymerase. Thermal protocol was: Initial denaturation at 95 °C (5 min); 40 cycles of 95 °C (1 min), 58 °C (0.5 min), and 72 °C (1 min); final elongation at 72 °C (5 min). PCR products were gel purified, ligated into pGEM-T (PROMEGA, Madison, WI, USA) and TOP10 competent cells were transformed. Four gene libraries were constructed (i.e., one each for root DNA, root cDNA; peat DNA, peat cDNA). Per gene library, plasmids were extracted from 96 clones, and inserts were Sanger sequenced (4 gene libraries x 96) at LGC genomics (Berlin, Germany). *nifH* sequences were clustered with JAGUC2 and OTUs were called at 97% sequence similarity^33^,^34^. Cluster representatives were phylogenetically affiliated with BLASTX^35^. 264 *nifH* genes and transcripts grouping into 2αTUs were recovered and are presented in the supporting information (Tables S3, S4). Diversity measures were calculated as described in Palmer *et al*.^34^. Coverages for all gene libraries were always >85%.

16S rRNA amplicons originating from RNA, were generated from roots and peat with primers 341F-785R as described^36^. Sequencing of the two amplicon libraries was done on the Illumina MiSeq V3 platform at LGC Genomics (Berlin, Germany) and approximately 13,000 quality-filtered reads were obtained per amplicon library. 16S rRNA derived sequences were analyzed with the QIIME pipeline^37^. In particular, OTUs were called at 97% sequence similarity and OTU representatives were aligned using PyNast. Chimeras were excluded uding ChimeraSlayer, and taxonomy was assigned to OTU representatives using RDP classifier.

## Results

Root, stem and leaf biomass of *L. fonkii* (n = 6) studied in Patagonia at both sites SKY and OBS amounted to means of 221 (range 79–367), 135 (range 49–225) and 119 (range 10–255) g d.w. m^−2^, respectively. Roots of *L. fonkii* had diameters of <1 mm and were densely covered by 15–20 nodules cm^−1^ (Fig. 1B,C). After incubation of *L. fonkii* from both sites with ^15^N_2_, fixed ^15^N was recovered in nodulated roots, stems, and leaf biomass, resulting in δ^15^N values of 4470 ± 1730, 3340 ± 1270, and 905 ± 245‰, respectively. Estimated BNF from ^15^N_2_ uptake into roots of intact plants were higher compared to rates obtained from incubation of cut roots (Table 2). Latter yielded about 70% of BNF in nodulated roots of control white clover plants (*Trifolium repens*) (Table 2). Ethylene production rates in cut roots of *L. fonkii* were about 8 times lower than ethylene production [i.e. acetylene reduction assay (ARA) as a proxy for nitrogenase activity] of *T. repens* cut roots. On the other hand, ethylene production rates of intact *L. fonkii* plants were similar to rates of cut roots. Both N_2_ fixation and ethylene production rates were lowest for root-free peat from 20 cm depth.

**Figure 2:**
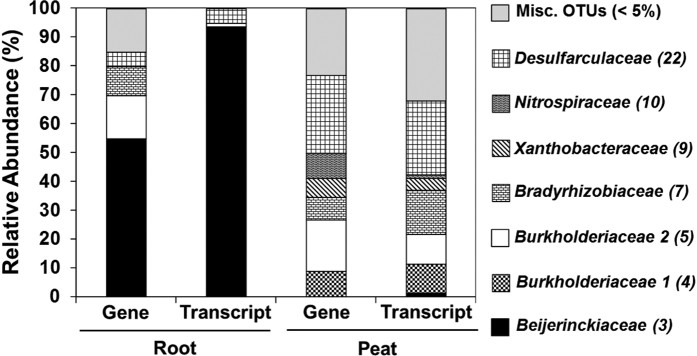
Relative abundances of *nifH* genes and transcripts in gene libraries retrieved from live *Lepidothamnus fonkii* roots and peat material. 264 *nifH* sequences grouping into 22 operational taxonomic units (OTUs) were recovered. OTUs with a relative abundance of >5% in one of the libraries are given. OUT numbers are shown in parentheses and related to *nifH* from the family-level taxa as indicated. Misc. OTUs (<5%): miscellaneous OTUs, relative abundance <5%. OTU 3 is associated with roots rather than with peat. (See also Tables S3, S4).

Scanning and transmission electron microscopy of roots or root thin sections indicated a high abundance of encapsulated, gram-negative bacteria with lipoid bodies and intact outer and inner membranes in presumably dead, peripheral cells of the nodules (Fig. 1C–F) of *L. fonkii* roots. Capsules and encapsulated cells resembled morphologies of *Beijerinckia* sp. grown in nitrogen free medium^38^. Nitrogenase encoding *nifH* genes and transcripts were diverse in peat and included operational taxonomic units (OTUs) related to *Rhizobiales, Burkholderiales*, and *Desulfarculales* (Fig. 2, Supplementary Tables S3, S4). Gene and transcript libraries of roots rather than those of peat were predominated by OTU3 that is closely related to *nifH* from *Beijerinckiaceae* (Supplementary Tables S3, S4). The relative abundance of OTU3 transcripts in root libraries was >93%, which indicates active N_2_ fixation by essentially one dominating group of root nodule-associated microbes. 16S rRNA analysis likewise revealed *Beijerinckiaceae* associated with roots rather than peat (Supplementary Figure S1).

## Discussion

Our finding of BNF, indicated by both ^15^N_2_ uptake and ARA, refute the common view that the BNF activity of *Podocarpaceae* is related to unspecific diazotrophs in the rhizosphere^11^,^13^. Although there is no endophytic symbiosis, electron microscopy and *nifH* transcripts indicated abundant living gram-negative bacteria in peripheral dead cells of root nodules of *L. fonkii*. Interestingly, nodulated roots of *L. fonkii* displayed similar rates of BNF as roots of a model plant with typical symbiotic N_2_ fixation by diazotrophic *Rhizobia* in nodules (Table 2; ref. 39). N_2_ fixation and ethylene production rates of other *Podocarpaceae* species, such as *P. lawrencei*^7^ were substantially lower than, but for *P. rospigliosii*^6^ of similar magnitude as those of *L. fonkii* roots when calculated on an entire root basis (Table 1). C_2_H_4_/^15^N_2_ ratios of 1.9–4.9 for *L. fonkii* fell in a range typically observed for most important nitrogenases^40^. We cannot exclude that a contamination of the applied ^15^N_2_ gas with ^15^NH_3_ or ^15^NO_x_ resulted in an overestimation of the BNF rate^25^. This bias, however, is likely small in our case, considering comparably high ^15^N enrichment, the acetylene reduction rates and respective C_2_H_4_/^15^N_2_ ratios observed for nodulated roots of *T. repens,* which compared well to values reported for *T. pratense*^41^. A significant contamination of the applied ^15^N_2_ gas and absorption of ^15^NH_3_ or ^15^NO_x_ could have led to lower C_2_H_4_/^15^N_2_ ratios and to an overestimation of N_2_ fixation rates of about 13%.

Nitrogenase genes (*nifH*) affiliating with *Bradyrhizobiaceae* and *Burkholderiaceae* were associated with peat and to some extent in *L. fonkii* roots (Fig. 2). Such diazotrophs were also detected in other studies on root-associated BNF of trees, but the diversity of active diazotrophs was much higher in these studies, indicating a non-specific association of diazotrophs with tree roots^42^,^43^. In our study, however, the expression of *Bradyrhizobiaceae* and *Burkholderiaceae* related *nifH* was essentially only detected in peat, indicating that free-living *Bradyrhizobiaceae* and *Burkholderiaceae* contribute to BNF in peat rather than to BNF in root nodules of *L. fonkii*. Accordingly, we assume that these taxa make a substantial contribution to BNF in the peat at our study sites. As *Bradyrhizobiaceae* and *Burkholderiaceae* are abundant taxa in peatlands or in other acidic and organic matter rich soils^44^,^45^,^39^, they could be key organisms for BNF in N limited ecosystems (see discussion below).

Despite the relatively small importance for BNF in root nodules *of L. fonkii*, active *Bradyrhizobiaceae* colonized roots of *L. fonkii* rather than peat as indicated by 16 rRNA amplicon sequencing (Fig. S1). Rhizobial Nod factors were reported to suppress plant innate immune response in nonlegumes^46^. Thus, it is tempting to speculate that initial colonization of roots by *Bradyrhizobiaceae* might enable subsequent colonization of other microbes.

The predominant and active N_2_ fixing, gram-negative bacteria of the *Beijerinckiaceae* identified in root nodules of *L. fonkii* (Figs 1 and 2) are well known as free-living diazotrophic bacteria that occur in water and soil including the rhizosphere of acidic peat soils^38^,^47^. *Beijerinckiaceae*-like *nifH* genes were recently also detected in association with *Sphagnum* mosses in an alpine bog^48^. Microbial diazotrophy accounted for most of the new N input associated with *Sphagnum* mosses^17^. Thus, plant associated diazotrophy was until recently underrated in bogs and in particular the role of diazotrophic *Beijerinckiaceae* may merit further attention.

Our study provides evidence that *L. fonkii* root associated, active diazotrophs fix substantial amounts of atmospheric N_2_. ^15^N_2_ enrichment in stems and leaf biomass supports significant and rapid translocation of fixed N excreted or leaking from diazotrophs in the root nodules to aboveground tissues (Table 2). Our results strongly support that this occurs in a specific association with *Beijerinckiaceae* in nodulated roots and refutes that reported N_2_ fixation by *Podocarpaceae* may only result from the activity of free-living bacteria in the rhizosphere^13^. Observed ranges of natural abundance of ^15^N in other *Podocarpaceae*, e.g. δ^15^N of −8 to −3‰ for *P. hallii* and *P. urbanii*^49^,^50^, suggest that such associated nitrogen fixation is certainly not a general feature of *Podocarpaceae*, however, or at least its contribution may not always be significant. For *L. fonkii*, specifically the following findings support an effective and specific association: (i) high N_2_ fixation rates, confirmed by both ^15^N_2_ uptake and active acetylene reduction (ii) molecular evidence of nitrogenase gene expression predominated by *Beijerinckiaceae* compared to a diverse diazotroph community in the surrounding rhizopheric peat soil (Fig. 2), and (iii) electron-microscopic images showing encapsulated bacteria with *Beijerinckiaceae*-like morphology densely colonizing the peripheral dead cell tissue of nodules (Fig. 1). Intact outer and inner cell membranes and lipoid bodies possibly consisting of poly-β-hydroxybutyra*te* (PHB) are typical for the gram-negative *Beijerinckiaceae*, further consolidating the conclusion that active *Beijerinckiaceae* reside inside nodules^51^. Obviously, peripheral dead cells of root nodules represent a favorable habitat that allows specific colonization and growth by *Beijerinckiaceae*. The high energy demand for N_2_ fixation and nutrients for growth may arise from enzymatic decay of plant cell compounds and dead bacterial cells. The layered ultrastructure of *L. fonkii* nodules (Fig. 1) suggests that *Beijerinckiaceae* are supported by continuous segregation of plant cells. High nitrogenase and BNF activities are in agreement with the formation of capsules (Fig. 1) that protects the oxygen sensitive nitrogenase^38^,^52^. The widespread occurrence of *Beijerinckiaceae* in a wide range of soils including those of low pH and high C-to-N ratio, their occurrence in the rhizoplane, and their well-recognized role as plant growth promoting bacteria might suggest a broad relevance for N-input of N-limited systems^53^,^54^. Thus, the proposed mechanism of plant-microbe interaction via necrosis of root cells and N-transfer from living bacteria to the host might represent an early variant of symbiotic diazotrophy and deserves more attention in future studies.

Indeed, evidence from growth experiments with the model diazotroph *Azotobacter vinelandii* suggests that ammonia could be excreted by or leak out of actively nitrogen fixing cells and can thus be easily transferred to the plant^55^. In this latter study, *A. vinelandii* accumulated up to 50 μM of ammonium and was capable of supporting algal growth in N-free medium. Although ^15^N recovered in leaf biomass might partly arise from a contribution of foliar endophytic nitrogen fixation, as reported for *Pinus flexilis*^56^, a translocation of N from nodules to leaves seems more likely due to higher ^15^N enrichment in the stem compared to leaf biomass.

A dense root biomass further indicates that *L. fonkii* can play a prominent role in the N cycle of south Patagonian bogs. Keeping in mind the limitation of our laboratory approach and inherent uncertainties in an extrapolation to field conditions, the potential N_2_ fixation is 13 mg N m^−2^ d^−1^ for the two study sites based on live root biomass, stems, leaves, and an incubation temperature of 15 °C. We cannot exclude overall smaller and seasonal different N_2_ fixation rates under *in-situ* conditions. Greater photosynthetically active radiation may improve the growth of root nodules and thus provides more niches for diazotrophs during the growing season. Lower *in-situ* temperatures would particularly limit the activity of diazotrophs in the early growing season. Further, we cannot exclude that preparation of *L. fonkii* altered the efficiency of N_2_ fixation during the incubation. Despite the methodological limitations, it seems that N_2_ fixation in root nodules of *L. fonkii* is one potent strategy of N acquisition in Patagonian bogs.

Considering recent studies on N_2_ fixation, plants and diazotrophs evolved different strategies to overcome N deficiency in ombrotrophic peatlands. Other pathways of N_2_ fixation include cyanobacteria, free-living diazotrophs or bryophyte-associated diazotrophs^17^,^19^,^28^,^48^,^57^, highlighting the diversity and niches of diazotrophs in ombrotrophic bogs. Further pathways of N acquisition, e.g. insect prey of *Drosera* sp.^58^, have also not yet been fully evaluated.

Comparing the BNF rates in other microhabitats, it seems that *L. fonkii* root nodules specifically colonized by diazotrophic *Beijerinckiaceae* represent ‘hot spots’ of BNF and thus of N acquisition. Existence of such specific associations and other reported strategies of N fixation challenge the current view on BNF in peatlands and in *Podocarpaceae*.

## Additional Information

**Accession codes:** Sequences of *nifH* were deposited at the *European Molecular Biology Laboratory* (*EMBL; www.ebi.ac.uk*) under accession numbers LT221262-LT221526. Illumina 16S rRNA amplicon sequences were deposited at GenBank’s short reads archive under the following accession numbers: SRA accession, SRP073705; BioProject ID, PRJNA319299; BioSamples SAMN04884733, SAMN04884734, SAMN04884735, and SAMN04884736.

**How to cite this article:** Borken, W. *et al*. Associative nitrogen fixation in nodules of the conifer *Lepidothamnus fonkii (Podocarpaceae)* inhabiting ombrotrophic bogs in southern Patagonia. *Sci. Rep.* **6**, 39072; doi: 10.1038/srep39072 (2016).

**Publisher's note:** Springer Nature remains neutral with regard to jurisdictional claims in published maps and institutional affiliations.

## Supplementary Material

Supplementary Information

## Figures and Tables

**Table 1:** Nitrogen fixation in nodules and nodulated roots of *Podocarpus* species.

Species	(nmol N g^−1^ d^−1^ nodules)	(nmol N g^−1^ d^−1^ nodulated roots)
*P. lawrencei*^7^	1128^†^	429^*,†^
*P. rospigliosii*^6^	3143^†^	1194^*,†^
*P. totara*^11^		3–7^‡^
*P. macrophyllus*^10^		*720*^†,‡^
*P. latifolius*^9^		36^†,‡^
*L. fonkii*^§^		2470

Literature data were recalculated to nodulated roots and for dry weight to allow for comparison.

*assuming 38% nodule mass per root mass^7^; †assuming 10% dry matter per fresh weight; ‡assuming a conversion factor of acetylene reduction to nitrogen fixation of 2; ^§^This study, root samples only.

**Table 2:** N_2_ fixation and ethylene (C_2_H_4_) production rates in *Lepidothamnus fonkii*.

	N_2_ fixation (μmol N g^−1^ d.w. d^−1^)	C_2_H_4_ production (μmol C_2_H_4_ g^−1^ d.w. d^−1^)	C_2_H_4_/N_2_
*L. fonkii* (entire plants)*, site OBS	1.02 ± 0.27	2.43 ± 0.45	4.90 ± 0.92
*L. fonkii* (entire plants)*, site SKY	1.25 ± 0.21	1.68 ± 0.25	2.69 ± 0.04
*L. fonkii*, intact roots†	3.09 ± 1.24	n.d.	n.d.
*L. fonkii*, cut roots‡	2.47 ± 0.28	2.35 ± 0.63	1.85 ± 0.79
*T. repens*, cut roots‡	3.94 ± 0.63	18.01 ± 5.27	9.07 ± 1.22
Root-free peat (0–10 cm)	1.69 ± 0.68	1.25 ± 0.32	1.67 ± 0.69

Mean ± s.d. *in-vitro* N_2_ fixation rates (determined by the ^15^N_2_ assay), unconverted C_2_H_4_ production rates (determined by the acetylene reduction assay), and ratios of C_2_H_4_ production to N_2_ fixation in entire plants of *L. fonkii* from OBS and SKY (n = 3), in nodulated roots of *L. fonkii* and *Trifolium repens* (white clover) and peat of 0–10 cm depth (all n = 6), n.d. = not detected.

^*^Incubation of intact plants and weighted average of N_2_ fixation in nodulated roots, stems and leaf biomass.

†N_2_ fixation of live roots, obtained from incubations of intact plants.

‡incubation of freshly cut, nodulated roots.
