# Supplementary material for: Associative nitrogen fixation in nodules of the conifer Lepidothamnus fonkii (Podocarpaceae) inhabiting ombrotrophic bogs in southern Patagonia
Source: Sci Rep. 2016 Dec 15;6:39072. doi: 10.1038/srep39072 (PMC5157042; doi:10.1038/srep39072)
Supplement: Supplementary Information [file srep39072-s1.pdf]

Supporting Information

Article title: Associative nitrogen fixation in nodules of the conifer *Lepidothamnus fonkii* *(Podocarpaceae)* inhabiting ombrotrophic bogs in southern Patagonia

Authors: Werner Borken, Marcus A. Horn, Stefan Geimer, Nelson A. Bahamonde Aguilar, Klaus-Holger Knorr

The following Supporting Information is available for this article:

Fig. S1 Relative abundances of 16S rRNA derived, classified operational taxonomic units (OTUs) in amplicon Illumina libraries retrieved from live roots of *Lepidothamnus fonkii* and peat material.

Table S1 Peat characteristics of the sampling sites Seno Skyring (SKY) and Seno Obstruccion (OBS)

Table S2 ^{15}N natural abundance of plant and peat reference material used for ^{15}N uptake calculations after conversion into ^{15}N atom %.

Table S3 Diversity of *nifH* genes and transcripts recovered from live roots of *Lepidothamnus fonkii* and peat material.

Table S4 Relative abundance of *nifH* gene and transcript (mRNA) defined OTUs retrieved from live roots of *Lepidothamnus fonkii* and peat material.

Fig. S1 Relative abundances of 16S rRNA derived, classified operational taxonomic units (OTUs) in amplicon Illumina libraries retrieved from live roots of *Lepidothamnus fonkii* and peat material. Approximately 13.000 quality-filtered reads were obtained per amplicon library. OTUs with a relative abundance of > 1% in one of the libraries are presented. *Beijerinckiaceae* include nitrogen fixing genera and are associated with *L. fonkii* roots rather than with peat material.

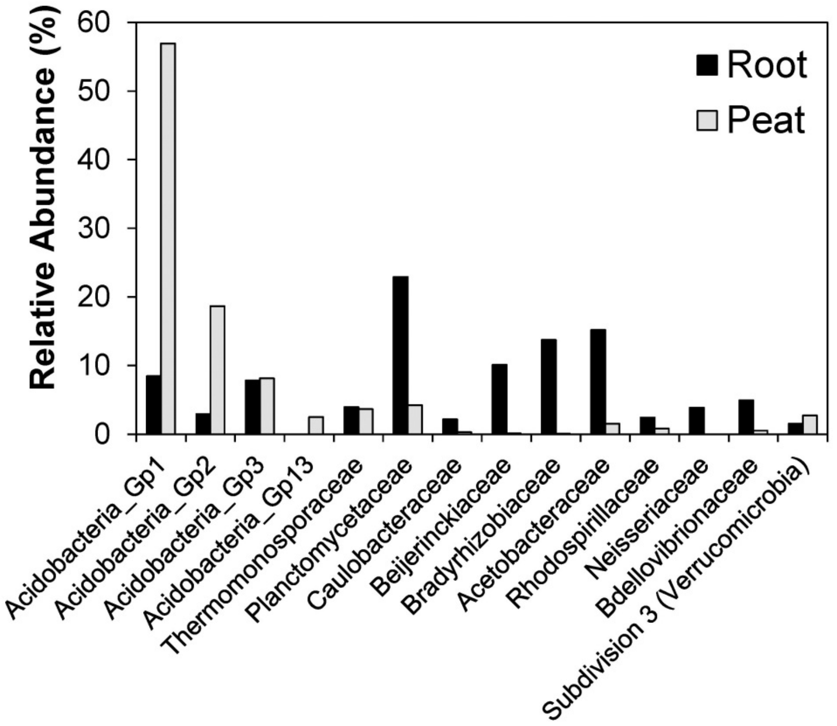

Table S1 Peat characteristics of the sampling sites Seno Skyring (SKY) and Seno Obstruccion (OBS): Content of nitrogen, molar ratios of carbon to nitrogen and ^{15}N isotopic signatures of bulk peat for the upper 80 cm.

Depth (cm)	OBS			SKY		
	N (%) by mass	Molar C/N ratio	$\delta^{15}N$ vs. air (‰)	N (%) by mass	Molar C/N ratio	$\delta^{15}N$ vs. air (‰)
0 - 10	0.69	93.9	-5.3	0.72	68.4	-4.4
10 - 20	0.70	92.0	-2.8	1.74	33.6	0.3
20 - 30	1.44	45.6	0.7	2.03	27.9	2.5
30 - 40	1.67	38.0	1.7	2.16	27.0	2.8
40 - 50	1.66	36.2	3.9	1.85	30.1	2.4
50 - 60	1.96	32.3	3.6	0.89	35.4	1.3
60 - 70	1.36	44.8	2.1	1.14	42.0	0.4
70 - 80	1.72	37.7	1.0	1.31	46.0	0.3

Table S2 ^{15}N natural abundance of plant and peat reference material used for ^{15}N uptake calculations after conversion into ^{15}N atom %. All reference plants and peat samples were obtained in close vicinity and similar microforms as of samples used for incubations. Peat samples were obtained as a representative sample from 0-10 cm depth.

	OBS		SKY	
	$\delta^{15}N$ (‰)	n	$\delta^{15}N$ (‰)	n
L. fonkii				
Leafs	-8.12 ± 0.83	3	-6.99 ± 0.77	3
Stems	-2.96 ± 1.44	3	-1.04 ± 0.75	3
Roots	-4.56 ± 0.95	3	-2.93 ± 0.65	3
Peat				
Root free bulk	-1.69 ± 0.21	6	+0.22 ± 1.71	6
T. repens				
Roots	-0.73 ± 0.45	3		

Table S3 Diversity of *nifH* genes and transcripts recovered from live roots of *Lepidothamnus* sp. and peat material.

Sample	No. of sequences	Good's coverage (%) *	No. of OTUs observed	No. of OTUs estimated †	*H* ‡	*E* §
Root						
Genes	20	86	7	8	1.1	0.7
Transcripts	76	99	3	3	0.3	0.3
Peat						
Genes	90	93	18	26	1.5	0.6
Transcripts	78	94	19	20	1.4	0.6

*Percent library coverage C = (1 - ns/nt) * 100 (n_s = OTUs that occur only once, n_t = total number of sequences)

†Chao1 richness estimate with upper and lower 95% confidence intervals given in parentheses.

‡Shannon diversity index.

§Species Evenness.

Table S4. Diversity of *nifH* genes and transcripts recovered from live roots of *Lepidothamnus fonkii* sp. and peat material.

Phylogenetic affiliation of close relative (taxonomically validated)	OTU	NifH of close taxonomically validated relative (Accession number)	*I* * (%)	NifH with highest *I* (Accession number)	*I (%)*	Relative abundance (%)			
						Root		Peat	
						Genes	mRNA	Genes	mRNA
Proteobacteria									
Alphaproteobacteria									
Rhizobiales									
Biejerinckiaceae	3	*Beijerinckia indica* subsp. *indica* ATCC 9039 (AJ563938)	97	*Beijerinckia indica* subsp. *indica* ATCC 9039 (AJ563938)	97	55.0	93.5	0.0	1.3
Bradyrhizobiaceae	7	*Bradyrhizobium elkanii* strain S 127 (DQ485702)	94	Uncultured bacterium clone JSS4-52 (HM750468)	96	10.0	0.0	7.8	15.4
Hyphomicrobiaceae	19	*Rhodomicrobium vannielii* ATCC 17100 (NC_014664)	86	Uncultured bacterium clone P1_A05 (GU945902)	91	5.0	0.0	0.0	0.0
Xanthobacteraceae	9	*Azorhizobium caulinodans* strain DSM 5975 (AJ563960)	92	Uncultured nitrogen-fixing bacterium clone SB-12 (EU090266)	95	0.0	0.0	6.7	3.8
Rhodospirillales									
Rhodospirillaceae	2	*Azospirillum brasilense* Sp245 (NC_016617)	95	Unidentified bacterium *nif* gene cluster (AF194084)	96	0.0	0.0	3.3	1.3
	8	*Azospirillum lipoferum* strain 4B (FQ311868)	95	*Azospirillum lipoferum* strain 4B (FQ311868)	95	5.0	0.0	1.1	9.0
Betaproteobacteria									
Burkholderiales									
Burkholderiaceae	4	*Burkholderia vietnamiensis* strain MMi-302 (EF158809)	88	Uncultured bacterium clone Yushu-14 (AY601050)	89	0.0	0.0	8.9	10.3

	5	*Burkholderia tropica* strain Aa1 (AY313947)	96	Uncultured bacterium clone Yushu-14 (AY601050)	96	15.0	1.3	17.8	10.3
Incertae sedis	6	*Leptothrix cholodnii* strain SP-6 (NC_010524)	95	*Leptothrix cholodnii* strain SP-6 (NC_010524)	95	0.0	0.0	0.0	1.3
Gammaproteobacteria									
Methylococcales									
Methylococcaceae	11	*Methylovulum miyakonense* HT12 (NZ_KB913025)	92	Uncultured bacterium clone pCOFnifE11 (EU156126)	94	0.0	0.0	3.3	2.6
Thiotrichales									
Thiotrichaceae	12	*Thiothrix nivea* DSM 5205 (NZ_JH651384)	87	Uncultured bacterium clone JSS16-2 (HM750519)	91	0.0	0.0	2.2	1.3
Deltaproteobacteria									
Desulfarculales									
Desulfarculaceae	20	*Desulfarculus baarsii* DSM 2075 (NC_014365)	89	Uncultured bacterium clone cloC-08 (JX268325)	99	0.0	0.0	1.1	0.0
	22	*Desulfarculus baarsii* DSM 2075 (NC_014365)	83	Uncultured nitrogen-fixing bacterium clone SB-24 (EU090278)	94	5.0	5.2	26.7	25.6
Desulfovibrionales									
Desulfovibrionaceae	18	*Desulfovibrio magneticus* MBC34 (NZ_ALAO01000346)	88	Uncultured nitrogen-fixing bacterium clone SB-37 (EU090291)	96	0.0	0.0	4.4	1.3
Syntrophobacterales									
Syntrophobacteraceae	14	*Syntrophobacter fumaroxidans* MPOB (NC_008554)	93	Uncultured bacterium clone SN1P1047 (JX154753)	94	0.0	0.0	1.1	1.3
Nitrospirae									

Nitrospirales									
Nitrospiraceae	1	*Thermodesulfovibrio yellowstonii* DSM 11347 (NC_011296)	93	*'Bradyrhizobium japonicum'* clone nifH 1-60 (GQ289578)	95	0.0	0.0	1.1	3.8
	10	*Thermodesulfovibrio yellowstonii* DSM 11347 (NC_011296)	87	'Bradyrhizobium japonicum' clone nifH 1-60 (GQ289578)	94	0.0	0.0	8.9	1.3
Chlorobi									
Chlorobia									
Chlorobiales									
Chlorobiaceae	15	*Chloroherpeton thalassium* ATCC 35110 (NC_011026)	91	*Chloroherpeton thalassium* ATCC 35110 (NC_011026)	91	0.0	0.0	1.1	1.3
	16	*Chlorobium phaeovibrioides* DSM 265 (NC_009337)	89	Uncultured nitrogen-fixing bacterium clone SB-38 (EU090292)	98	0.0	0.0	1.1	2.6
Spirochaetes									
Spirochaetales									
Spirochaetaceae	21	*Treponema azotonutricium* ZAS-9 (NC_015577)	90	Uncultured bacterium clone pTKY2312_10 (JN217110)	92	0.0	0.0	1.1	0.0
Verrucomicrobia									
Opitutae									
Puniceicoccales									
Puniceicoccaceae	13	*Coraliomargarita akajimensis* DSM 45221 (CP001998)	90	Uncultured bacterium clone JSS4-10 (HM750471)	91	0.0	0.0	0.0	2.6
	17	*Coraliomargarita akajimensis* DSM 45221 (CP001998)	94	*Opitutaceae* bacterium TAV5 (AGJF01000003)	98	5.0	0.0	2.2	3.8

[*] Identity of in silico translated nucleic acid sequences as determined by BLASTX analysis.
